# Susceptibility Trends of Zoliflodacin against Multidrug-Resistant *Neisseria gonorrhoeae* Clinical Isolates in Nanjing, China, 2014 to 2018

**DOI:** 10.1128/AAC.00863-20

**Published:** 2021-02-17

**Authors:** Wenjing Le, Xiaohong Su, Xiangdi Lou, Xuechun Li, Xiangdong Gong, Baoxi Wang, Caroline A. Genco, John P. Mueller, Peter A. Rice

**Affiliations:** a STD Clinic, Institute of Dermatology, Chinese Academy of Medical Sciences and Peking Union Medical College, Nanjing, China; b Department of Dermatology, Plastic Surgery Hospital, Chinese Academy of Medical Sciences and Peking Union Medical College, Beijing, China; c Department of Immunology, School of Medicine, Tufts University, Boston, Massachusetts, USA; d Entasis Therapeutics, Waltham, Massachusetts, USA; e Division of Infectious Diseases and Immunology, University of Massachusetts Medical School, Worcester, Massachusetts, USA

**Keywords:** *N. gonorrhoeae*, DNA gyrase, zoliflodacin, susceptibility

## Abstract

Previously, we reported the potent activity of a novel spiropyrimidinetrione, zoliflodacin, against Neisseria gonorrhoeae isolates collected in 2013 from symptomatic men in Nanjing, China. Here, we investigated trends of susceptibilities to zoliflodacin in 986 isolates collected from men between 2014 and 2018. N. gonorrhoeae isolates were tested for susceptibility to zoliflodacin and seven other antibiotics. Mutations in the *gyrA*, *gyrB*, *parC*, *parE*, and *mtrR* genes were determined by PCR and sequencing. The MICs of zoliflodacin ranged from ≤0.002 to 0.25 mg/liter; the overall MIC_50_ and MIC_90_ were 0.06 mg/liter and 0.125 mg/liter, respectively, in 2018, increasing 2-fold from 2014. However, the percentage of isolates with lower zoliflodacin MICs declined in each year sequentially, while the percentage with higher MICs increased yearly (*P* ≤ 0.00001). All isolates were susceptible to spectinomycin but resistant to ciprofloxacin (MIC ≥ 1 mg/liter); 21.2% (209/986) were resistant to azithromycin (≥1 mg/liter), 43.4% (428/986) were penicillinase-producing N. gonorrhoeae (PPNG), 26.9% (265/986) were tetracycline-resistant N. gonorrhoeae (TRNG), and 19.4% (191/986) were multidrug-resistant (MDR) isolates. 202 isolates with the lowest (≤0.002 to 0.015 mg/liter) and highest (0.125 to 0.25 mg/liter) zoliflodacin MICs were quinolone resistant with double or triple mutations in *gyrA*; 193/202 (95.5%) also had mutations in *parC*. There were no D429N/A and/or K450T mutations in GyrB identified in the 143 isolates with higher zoliflodacin MICs; an S467N mutation in GyrB was identified in one isolate. We report that zoliflodacin continues to have excellent *in vitro* activity against clinical gonococcal isolates, including those with high-level resistance to ciprofloxacin, azithromycin, and extended-spectrum cephalosporins.

## Abstract (precis)

Previously, we reported the potent activity of a novel spiropyrimidinetrione, zoliflodacin, against Neisseria gonorrhoeae isolates collected in 2013 from symptomatic men in Nanjing, China. Here, we investigated trends of susceptibilities to zoliflodacin in 986 isolates collected from men between 2014 and 2018. N. gonorrhoeae isolates were tested for susceptibility to zoliflodacin and seven other antibiotics.

## INTRODUCTION

Neisseria gonorrhoeae, the causative agent of the sexually transmitted infection gonorrhea, has developed resistance to all previously recommended antimicrobial agents for treatment, including sulfonamides, penicillins, tetracyclines, and fluoroquinolones (1). Currently, dual antimicrobial therapy with ceftriaxone at 250 mg or cefixime at 400 mg plus azithromycin at 1 g is recommended as the first-line treatment for uncomplicated gonorrhea by the World Health Organization (WHO) (2), and therapy with ceftriaxone 500 mg intramuscular alone, without azithromycin, is recommended by the U.S. Centers for Disease Control and Prevention (CDC) (3). Resistance to extended-spectrum cephalosporins (ESCs) and azithromycin is increasing worldwide. Gonococcal isolates with decreased susceptibility to cefixime and/or ceftriaxone have been reported in China (4), Japan (5), Australia (6), European countries (7), and the United States (8), and isolates with high-level resistance to ceftriaxone have been identified in Japan, Australia, France, Spain, Denmark, Canada, Ireland, and China (9–11). The reported prevalences of azithromycin-resistant N. gonorrhoeae isolates are 18.6% in China (4), 14.5% in Japan (5), 6.2% in Australia (6), 7.5% in 25 European countries (7), 4.6% in the United States (8), and 6.1% in western Africa (12). The first documented case that failed treatment with the recommended dual therapy was reported from the United Kingdom in 2016 (13), and the first gonococcal isolates (the A2543 clone) with combined ceftriaxone resistance plus high-level azithromycin resistance were identified in the United Kingdom (14) and Australia (15) in 2018.

Increased antimicrobial resistance (AMR) in N. gonorrhoeae poses an emerging global public health threat of untreatable gonococcal infections. New oral antimicrobial agents with activity against N. gonorrhoeae are needed urgently. The WHO includes N. gonorrhoeae on its list of “priority pathogens” that require new antibiotics for treatment (16), and the U.S. CDC has designated drug-resistant N. gonorrhoeae an urgent threat (17). Zoliflodacin (also known as AZD0914 and ETX0914) is a novel spiropyrimidinetrione bacterial DNA gyrase/topoisomerase inhibitor with broad-spectrum *in vitro* activity against Gram-positive and fastidious Gram-negative organisms, including N. gonorrhoeae (18, 19). A recent multicenter, randomized, phase 2 clinical trial demonstrated that zoliflodacin was effective in treating gonococcal urogenital and rectal infections and supports a larger, more definitive study of zoliflodacin for the treatment of uncomplicated gonorrhea (20). We showed previously that zoliflodacin was highly effective against clinical isolates of N. gonorrhoeae *in vitro*, including high-level ciprofloxacin-resistant and multidrug-resistant (MDR) isolates collected in 2013 in Nanjing, China (21). Here, *in vitro* activities and trends of zoliflodacin susceptibilities were determined for clinical gonococcal isolates (including multidrug-resistant isolates) collected between 2014 and 2018 in Nanjing. Mutations in the quinolone-resistance-determining regions (QRDRs) of the *gyrA*, *parC*, *gyrB*, *parE*, and *mtrR* genes were also determined for isolates across the zoliflodacin MIC distribution range.

## RESULTS

### Susceptibilities to zoliflodacin and other antimicrobials.

**FIG 1:**
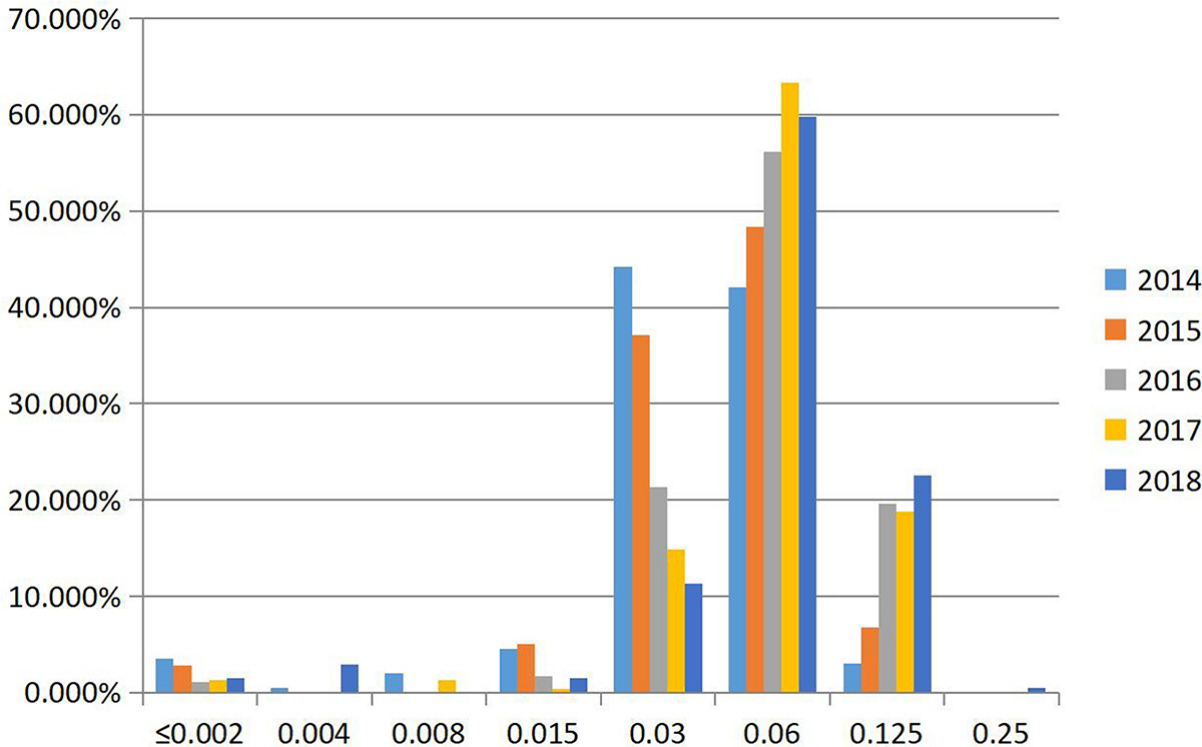
MIC distributions of zoliflodacin for 986 clinical N. gonorrhoeae isolates, 2014 to 2018.

Susceptibilities (MICs) of N. gonorrhoeae to zoliflodacin and seven antimicrobials previously or currently used for the treatment of gonorrhea are summarized for the 986 clinical isolates in Table 1. All isolates except one were inhibited by ≤0.125 mg/liter of zoliflodacin (the remaining isolate had an MIC of 0.25 mg/liter). MICs to zoliflodacin ranged from ≤0.002 to 0.25 mg/liter overall, with MIC_50_ and MIC_90_ values of 0.06 mg/liter and 0.125 mg/liter, respectively. One hundred forty-three (14.5%) isolates had zoliflodacin MICs at the upper end of the distribution range (0.125 to 0.25 mg/liter), and 59 (6%) isolates had MICs at the lower end of the MIC distribution range (≤0.002 to 0.015 mg/liter). The percentage of isolates with an MIC of 0.03 mg/liter to zoliflodacin declined in each year sequentially (χ^2^ = 82.237; *P* = 0.000), while the percentage with MICs of 0.06 and 0.125 mg/liter increased correspondingly (χ^2^ = 20.739 and 41.717, respectively; *P* ≤ 0.00001 by a chi-square test for linear trends), as shown in Fig. 1. Overall, the proportion of isolates with zoliflodacin MICs of 0.125 to 0.25 mg/liter increased from 3.1% (6/197) in 2014 to 23.0% (47/204) in 2018 (χ^2^ = 43.112; *P* < 0.0001).

**TABLE 1:** Susceptibilities and MICs of zoliflodacin and seven antimicrobials previously or currently used for the treatment of gonorrhea against 986 clinical N. gonorrhoeae isolates

Antimicrobial	No. (%) of isolates			MIC (mg/liter)		
	Susceptible	Intermediate	Resistant	Range	MIC_50_	MIC_90_
Zoliflodacin	986 (100)			≤0.002 to 0.25	0.06	0.125
Penicillin G	0	171 (17.3)	815 (82.7)	0.125 to ≥16	4	≥16
Tetracycline	4 (0.4)	150 (15.2)	832 (84.4)	≤0.125 to ≥32	2	≥32
Ciprofloxacin	0	0	986 (100)	1 to ≥16	≥16	≥16
Azithromycin	551 (55.9)	226 (22.9)	209 (21.2)	≤0.015 to ≥2,048	0.5	4
Spectinomycin	986 (100)	0	0	≤4 to 32	32	32
Cefixime	948 (96.1)		38 (3.9)	≤0.002 to >2	0.03	0.25
Ceftriaxone	984 (99.8)		2 (0.2)	≤0.002 to 1	0.03	0.125

All 986 isolates were resistant to ciprofloxacin; 777 (78.8%) showed high-level resistance (≥16 mg/liter) (22). During the 5-year study period, the annual percentage of ciprofloxacin-resistant isolates at each MIC point (from 1 mg/liter to ≥16 mg/liter) did not shift in either direction in the 5-year period. MICs of gonococcal isolates for zoliflodacin were lower than those for ciprofloxacin (*P* < 0.0001), with a median difference of at least 267-fold. Four hundred twenty-eight isolates (43.4%) were penicillinase-producing N. gonorrhoeae (PPNG), and 265 (26.9%) were tetracycline-resistant N. gonorrhoeae (TRNG) isolates. The percentage of penicillin-resistant isolates increased from 70% to 86.3% over the 5 years (χ^2^ = 17.641; *P* < 0.0001). Although all isolates were susceptible to spectinomycin, the percentage of isolates with lower spectinomycin MICs (8 mg/liter and 16 mg/liter) declined (χ^2^ = 16.35 and 93.71; *P* = 0.0001 and *P* < 0.0001, respectively), while the percentage with higher MICs (32 mg/liter) increased over the 5 years (χ^2^ = 112.514; *P* < 0.0001).

Two hundred nine (21.2%) isolates were resistant to azithromycin (MIC ≥ 1 mg/liter), and 62 (6.3%) displayed high-level resistance (MIC ≥ 256 mg/liter). The percentage of isolates with lower azithromycin MICs (0.06 mg/liter and 0.125 mg/liter) increased over the 5 years (χ^2^ = 16.916 and 22.099, respectively; *P* < 0.0001), while the percentage with higher MICs (0.5 mg/liter and ≥1,024 mg/liter) declined yearly (χ^2^ = 15.403 and 12.268, respectively; *P* < 0.001). Overall, the percentage of azithromycin-resistant isolates (MIC ≥ 1 mg/liter) decreased from 27.9% to 15.2% over the 5 years, and the percentage of azithromycin-susceptible isolates increased from 72.1% to 84.8% (χ^2^ = 14.618; *P* < 0.001). One hundred fifty-eight isolates (15.2%) exhibited decreased susceptibility (MIC of 0.125 to 0.25 mg/liter [*n* = 156]) or resistance (MIC = 1 mg/liter [*n* = 2]) to ceftriaxone, and 102 isolates (10.1%) displayed decreased susceptibility (MIC of 0.25 mg/liter [*n* = 64]) or resistance (MIC of 0.5 mg/liter [*n* = 36] and MIC of >2 mg/liter [*n* = 2]) to cefixime. The percentage of isolates with lower ceftriaxone MICs (≤0.03 mg/liter) declined in each year sequentially (χ^2^ = 10.512; *P* < 0.01), while the percentage with higher MICs (0.06 mg/liter and 0.125 mg/liter) increased yearly (χ^2^ = 10.18 and 4.231; *P* < 0.01 and *P* < 0.05, respectively). The percentage of isolates with lower cefixime MICs (0.015 mg/liter and 0.03 mg/liter) declined (χ^2^ = 23.324 and 10.734; *P* < 0.001 and *P* < 0.01, respectively), while the percentage with higher MICs (0.06 to 0.5 mg/liter) increased over the 5 years (χ^2^ = 8.68, 14.683, 5.042, and 20.056; *P* values of <0.01, <0.001, <0.05, and <0.0001, respectively). One hundred ninety-one (19.4%) isolates showed multidrug resistance (MDR). The proportion of MDR isolates increased from 7.1% in 2014 to 27% in 2016 and then decreased to 21.1% in 2018 (χ^2^ = 12.82; *P* = 0.00034). The two MDR isolates with high-level resistance to ceftriaxone (MIC of 1.0 mg/liter), cefixime (MIC ≥ 2.0 mg/liter), ciprofloxacin (MIC ≥ 16 mg/liter), penicillin (MIC of 4 mg/liter), and tetracycline (MIC of 4 mg/liter) had low zoliflodacin MIC values (0.03 and 0.06 mg/liter, respectively).

### Characterization of amino acid substitutions in GyrA, GyrB, ParC, and ParE.

All 202 isolates tested were ciprofloxacin resistant (MICs of 2 to ≥16 mg/liter). All isolates had double or triple mutations in the *gyrA* gene. Both the S91F and D95A/G/N/Y amino acid substitutions in GyrA were identified in the 202 isolates. Sixteen (11.2%) of the isolates in the higher zoliflodacin MIC distribution group and two (3.4%) in the lower-MIC group also had an additional A92P amino acid substitution in GyrA. ParC substitutions were observed in 97.2% of the isolates in the higher zoliflodacin MIC distribution group and in 91.5% of the isolates in the lower-MIC group. Single, double, and triple ParC substitutions were identified in 114 (79.7%), 22 (15.4%), and 3 (2.1%) of the isolates in the higher-MIC distribution group and in 66.1%, 25.4%, and 0% of the isolates in the lower-MIC group, respectively. The amino acid substitution at position S87 in ParC, including S87C, S87I, S87N, or S87R, was present in 79.7% of isolates in the higher-MIC distribution group and in 81.4% of isolates in the lower-MIC group. The most common double substitutions in ParC were S87R plus S88P (10.7%) in the higher-MIC group and S87R plus G85D (15.3%) in the lower-MIC group. The three isolates in the higher-MIC group had the same triple substitutions (S87R, A123V, and A129V). The A89T, G120R, A123V, and A129V mutations in ParC are newly described here. GyrB substitutions/insertions were identified in four isolates (two with V470I substitutions, one with an S467N substitution, and one with an arginine [A] insertion at position 480 [480A]) at the upper end of the MIC distribution group, but none were identified in the low-MIC group. All four isolates with a GyrB mutation had MIC values of 0.125 mg/liter for zoliflodacin and 4 mg/liter or higher for ciprofloxacin. Amino acid substitutions in ParE were identified in 57 isolates (39.9%) in the high zoliflodacin MIC distribution group. The most common single substitution in ParE was D437N, which found more frequently in isolates with MICs at the upper end of the zoliflodacin MIC distribution range (23.1%) than in those with MICs at the lower end of the range (6.78%) (*P* < 0.01). The overall frequencies of amino acid substitutions in GyrA, GyrB, ParC, and ParE were no different across the MIC distribution range (Table 2).

**TABLE 2:** Comparison of amino acid substitutions in GyrA, GyrB, ParC, and ParE in isolates with lower zoliflodacin MICs versus isolates with higher MICs

Protein and amino acid substitution	No. (%) of N. gonorrhoeae isolates		*P* value^*c*^
	Lower zoliflodacin MIC group (*n* = 59)^*a*^	Higher zoliflodacin MIC group (*n* = 143)^*b*^	
GyrA	59 (100)	143 (100)	NA
S91F	59 (100)	143 (100)	
D95A/G/N/Y	59 (100)	143 (100)	
A92P	2 (3.39)	16 (11.19)	0.103
D80N	1 (1.69)	0	0.292
V81I	1 (1.69)	0	0.292

ParC	54 (91.53)	139 (97.20)	0.13
G85C/D/A	14 (23.73)	7 (4.90)	<0.001
D86N	3 (5.08)	20 (13.99)	0.088
S87C/I/N/R	48 (81.36)	114 (79.72)	0.943
S88P	1 (1.69)	10 (6.99)	0.181
A89T	1 (1.69)	1 (0.70)	0.499
E91G	2 (3.39)	7 (4.90)	1.000
G120R	0	2 (1.40)	1.000
A123V	0	3 (2.10)	0.557
A129V	0	3 (2.10)	0.557

GyrB	0	4 (2.80)	0.32
S467N	0	1 (0.70)	1.000
V470I	0	2 (1.40)	1.000
+480A	0	1 (0.70)	1.000

ParE	20 (33.90)	57 (39.86)	0.43
D437H/N	5 (8.47)	34 (23.78)	0.01
P456S	14 (23.73)	22 (15.38)	0.227
P469L	0	1 (0.70)	1.000
D425Y	1 (1.69)	0	0.292
L462I	1 (1.69)	0	0.292

a Isolates with zoliflodacin MICs of ≤0.002 to 0.015 mg/liter.

b Isolates with zoliflodacin MICs of 0.125 to 0.25 mg/liter.

c Determined by the χ^2^ or Fisher exact test. NA, not applicable.

### Mutations in *mtrR*.

A number of single or multiple mutations were identified in the 202 isolates with the lowest (≤0.002 to 0.015 mg/liter) and highest (0.125 to 0.25 mg/liter) zoliflocacin MICs. These included an adenosine (A) deletion in the *mtrR* promoter region and mutations in the *mtrR* coding region that resulted in amino acid changes in MtrR: A39T, A40D, G45D, F62L, D79N, T86A, H105Y, and E117K mutations, singly or in combination (see Table S2 in the supplemental material). A total of 175 (86.6%) isolates carried the A deletion: 48 (81.4%) in the low zoliflodacin MIC group and 127 (88.8%) in the high zoliflodacin MIC group (*P* = 0.2346). There were no significant differences in the rates of individual mutations (singly or combined) in MtrR accompanied (or not) by an A deletion in the promoter region, except for an H105Y mutation accompanied by an A deletion in the promoter, which accounted for 62.7% (37/59) of isolates with low zoliflodacin MICs and 41.3% (59/143) of isolates in the high zoliflodacin MIC group (*P* < 0.01) (Table S2).

## DISCUSSION

We determined susceptibility trends in the *in vitro* antibacterial activities of zoliflodacin and seven other antimicrobial agents against 986 clinical gonococcal isolates collected over a 5-year period (2014 to 2018). The 986 gonococcal isolates were susceptible to zoliflodacin, and all were resistant to ciprofloxacin. Nearly one-quarter of the isolates were resistant to azithromycin or were TRNG isolates. More than 40% were PPNG isolates, and just under 20% were MDR isolates. All 986 isolates had zoliflodacin MICs below the breakpoint (MIC ≥ 0.5 mg/liter) that has been proposed, guided by clinical efficacy (20). Similar to other reports (19, 23), zoliflodacin exhibited an MIC range of 0.002 to 0.25 mg/liter, and there was no correlation between zoliflodacin MICs at the upper end of the MIC range and ciprofloxacin resistance (19, 24, 25). Furthermore, zoliflodacin exhibited low MICs (0.03 and 0.06 mg/liter) in two isolates that were fully resistant to ceftriaxone and cefixime. A modest temporal shift in the MICs to zoliflodacin was observed over the 5-year period.

Zoliflodacin is a novel spiropyrimidinetrione bacterial DNA gyrase/topoisomerase inhibitor that prevents bacterial DNA biosynthesis and results in the accumulation of double-strand cleavages through a mechanism distinct from that in fluoroquinolones (1, 18, 24). In our study, all the ciprofloxacin-resistant zoliflodacin-sensitive isolates tested displayed double or triple mutations in GyrA; >90% had additional amino acid substitutions in ParC.

In contrast to fluoroquinolones, zoliflodacin inhibits the GyrB subunit of type II topoisomerase; specific mutations in GyrB can result in increased resistance to zoliflodacin (24, 25). We did not find mutations such as D429N, D429A, or K450T alterations in GyrB, which have been identified *in vitro* and select for resistant mutants that result in zoliflodacin MICs of 0.5 to 8 mg/liter (24, 25). However, we found that 4/143 (2.8%) gonococcal isolates at the upper end of the MIC distribution range (0.125 and 0.25 mg/liter) harbored a GyrB mutation; however, the amino acid substitutions/insertions (S467N, V470I, or 480A) were not associated with resistance. An S467N amino acid substitution in GyrB, which did not result in reduced susceptibility to zoliflodacin, has been reported in a clinical gonococcal isolate (19). V470I or 480A mutations have not been reported previously in clinical isolates or in *in vitro*-selected resistant mutants.

Mutations in *mtrR*, which result in the overexpression of the MtrCDE efflux pump, can increase the efflux of antimicrobials and reduce susceptibility to numerous antimicrobials (1, 26). The MtrCDE efflux pump can also influence susceptibility to zoliflodacin (25). Inactivation of the MtrCDE efflux pump has been shown to decrease the MIC of zoliflodacin in N. gonorrhoeae strain H041 from 0.125 to 0.004 mg/liter (25). In our study, an adenine (A) deletion in the *mtrR* promoter and a number of mutations in MtrR (or both) were identified in isolates that possessed either lower or higher zoliflodacin MICs. A single H105Y amino acid substitution was the most common substitution present in MtrR; this change was identified in 50% of the isolates. The single H105Y amino acid substitution, which lies outside the known DNA binding domain of MtrR, is generally thought not to be involved in the active repressor function of MtrR; it has also been shown to be associated with N. gonorrhoeae isolates that are fully sensitive to ceftriaxone (27). One possibility is that the H105Y mutation may interfere with MtrR dimerization, resulting in a reduction of MtrR binding to target sequences (28).

Few studies have examined the impact of *parE* mutations on quinolone resistance in N. gonorrhoeae (29, 30). Clinical gonococcal isolates with P439S amino acid substitutions in ParE did not result in a significant increase in the MIC to ciprofloxacin (30, 31). The clinical relevance of the ParE mutations identified in our study is unclear.

In conclusion, zoliflodacin demonstrated potent *in vitro* antibacterial activity against a recent collection of clinical gonococcal isolates from China (2014 to 2018), including isolates with high-level resistance to ciprofloxacin, azithromycin, and extended-spectrum cephalosporins. Zoliflodacin MICs shifted upward temporally in the 5-year period in the absence of clinical use. These results confirm the lack of preexisting clinical resistance to zoliflodacin. Continued monitoring of antimicrobial susceptibility to zoliflodacin, a promising new oral antibacterial agent, for the treatment of uncomplicated gonorrhea is warranted.

## MATERIALS AND METHODS

### Bacterial isolates.

From January 2014 to December 2018, a total of 986 gonococcal isolates were collected from male patients with symptomatic urethritis (urethral discharge and/or dysuria) attending the STD Clinic at the Institute of Dermatology, Chinese Academy of Medical Sciences, Nanjing, China. All men except one reported that they were heterosexual. Urethral exudates were collected with cotton swabs, immediately inoculated onto Thayer-Martin medium (Zhuhai DL Biotech, China), and cultured in candle jars at 36°C for 24 to 48 h. Gonococcal isolates were identified by colony morphology, Gram’s stain and oxidase testing, and growth on GC chocolate agar base (Difco, Detroit, MI) supplemented with 1% IsoVitaleX (Oxoid, USA). Gonococcal colonies were suspended in tryptone-based soy broth and frozen (−70°C) until used for antimicrobial testing.

### Antimicrobial susceptibility testing.

Zoliflodacin powder was provided by Entasis Therapeutics (Waltham, MA). The MICs (milligrams per liter) of N. gonorrhoeae isolates to zoliflodacin, penicillin, tetracycline, ciprofloxacin, spectinomycin, azithromycin, cefixime, and ceftriaxone were determined by the agar dilution method in accordance with Clinical and Laboratory Standards Institute (CLSI) guidelines (32). ATCC 49226 and WHO reference strains F, G, L, O, and P were used as quality controls (QCs). The MIC ranges of zoliflodacin for QC strain ATCC 49226 were 0.125 to 0.25 mg/liter in each antimicrobial susceptibility testing run in this study, in accordance with the defined MIC QC ranges (0.06 to 0.5 mg/liter) for zoliflodacin (33). Criteria for decreased susceptibility to ceftriaxone (MIC ≥ 0.125 mg/liter) and cefixime (MIC ≥ 0.25 mg/liter) were defined by the WHO (34). Using CLSI (32) and EUCAST (35) (for azithromycin only) criteria, the following MIC breakpoints were used to ascertain resistance: ≥128 mg/liter for spectinomycin, ≥2 mg/liter for penicillin and tetracycline, and ≥1 mg/liter for ciprofloxacin and azithromycin. The breakpoint for zoliflodacin of ≥0.5 mg/liter was utilized as previously described (20). Multidrug-resistant (MDR) N. gonorrhoeae was defined as decreased susceptibility or resistance to extended-spectrum cephalosporins (ESCs) plus resistance to at least two of the following antimicrobials: penicillin, ciprofloxacin, and azithromycin (36, 37).

### Identification of gene mutations that resulted in amino acid substitutions in GyrA, GyrB, ParC, and ParE.

One hundred forty-three gonococcal isolates with zoliflodacin MICs (0.125 mg/liter and 0.25 mg/liter) at the upper end of the MIC distribution range and 59 isolates with lower zoliflodacin MICs (≤0.002 to 0.015 mg/liter) were selected for genetic resistance determinant studies. Mutations in the quinolone-resistance-determining regions (QRDRs) of the *gyrA*, *gyrB*, *parC*, and *parE* genes were determined by PCR and DNA sequencing using primers described previously (38–40) (see Table S1 in the supplemental material). Genomic DNA was extracted from gonococcal isolates using a rapid bacterial genomic DNA isolation kit (DNA-EZ Reagents V All-DNA-Fast-Out; Sangon Biotech Co. Ltd., Shanghai, China). PCR amplification and sequencing of the genes were carried out by Nanjing Qingke Biotech Co. Ltd.

### Evaluation of mutations in the *mtrR* gene.

To identify mutations that potentially could cause enhanced expression of the MtrCDE efflux pump, mutations in the *mtrR* gene and promoter region were identified by PCR. Sequencing of *mtr* genes from 202 isolates was performed as described previously (27).

### Data analysis.

Chi-square (χ^2^) testing was used to compare the rates of resistance in different years, and a chi-square test for linear trends was used to assess the changes in the MICs and the proportion of isolates resistant to antibiotics. SPSS version 19.0 was used for statistical analysis; a *P* value of <0.05 was considered statistically significant.

## Supplementary Material

Supplemental file 1
