# Supplementary material for: Susceptibility Trends of Zoliflodacin against Multidrug-Resistant Neisseria gonorrhoeae Clinical Isolates in Nanjing, China, 2014 to 2018
Source: Antimicrob Agents Chemother. 2021 Feb 17;65(3):e00863-20. doi: 10.1128/AAC.00863-20 (PMC8092536; doi:10.1128/AAC.00863-20)
Supplement: Supplemental file 1 [file AAC.00863-20-s0001.pdf]

Supplemental Tables

Table 1 . Primers for detecting gonococcal *gyrA*, *gyrB*, *parC*, and *parE* genes

Target	Primer name	Primer Sequence(5’-3’)	Position	Reference
gyrA	gyrA-F	CGGCGCGTACTGTACGCGATGCA	160-438	1
	gyrA-R	AATGTCTGCCAGCATTTCATGTGAGA		
gyrB	NG-GYRB-A	ACTGCCAAGAAAAAGACCCT	1235-1508	2
	NG-GYRB-B	TCGGCATCGGTCATGATGAT		
parC	parC-F	ATGCGCGATATGGGTTTGAC	166-420	1
	parC-R	GGACAACAGCAATTCCGCAA		
parE	parE-1F	GCCGAACTCGCCATCCGTCAG	1156-1595	3
	parE-1R	GGGAAGTGGCGGTAGAACAGG		

1. Tanaka M, Nakayama H, Haraoka M, Saika T. 2000. Antimicrobial Resistance of *Neisseria gonorrhoeae* and High Prevalence of Ciprofloxacin-Resistant Isolates in Japan, 1993 to 1998. J Clin Microbiol 38(2): 521 – 525.
2. Deguchi T, Yasuda M, Nakano M, Ozeki S, Kanematsu E, Kawada Y, Ezaki T, Saito I. 1996. Uncommon Occurrence of Mutations in the gyrB Gene Associated with Quinolone Resistance in Clinical Isolates of *Neisseria gonorrhoeae*. Antimicrob Agents Chemother 40(10): 2437 – 2438.
3. Unemo M, Fasth O, Fredlund H, Limnios A, Tapsall J. 2009. Phenotypic and genetic characterization of the 2008 WHO *Neisseria gonorrhoeae* reference strain panel intended for global quality assurance and quality control of gonococcal antimicrobial resistance surveillance for public health purposes. J Antimicrob Chemother 63(6): 1142 – 1151.

Table 2. Comparison of mutations in the *mtrR* gene in isolates with lower zoliflodacin MICs versus isolates with higher MICs that resulted in amino acid substitutions in MtrR

mtrR mutations: in the promoter region and amino acid substitutions in MtrR	No.(%) of *N. gonorrhoeae* isolates		*P*-value[c]
	lower zoliflodacin MICs group (n=59)[a]	higher zoliflodacin MICs group (n=143)[b]	
WT	0	1 (0.70%)	1.000
A-deletion in the *mtr* promoter[d]	2 (3.39%)	18 (12.59%)	0.0669
A-deletion[d], A39T	1 (1.69%)	15 (10.49%)	0.0425
A-deletion[d], G45D	6 (10.17%)	28 (19.58%)	0.1559
A-deletion[d], T86A	0	1 (0.70%)	1.000
A-deletion[d], H105Y	37 (62.71%)	59 (41.26%)	0.008
A-deletion[d], A40D, T86A	0	2 (1.40%)	1.000
A-deletion[d], D79N,T86A	0	2 (1.40%)	1.000
A-deletion[d],D79N,T86A, H105Y	2 (3.39%)	2 (1.40%)	0.5818
A39T	2 (3.39%)	1 (0.70%)	0.2048
G45D	6 (10.17%)	7 (4.90%)	0.2828
A39T, F62L	2 (3.39%)	0	0.0843
A40D, T86A	0	5 (3.50%)	0.324
D79N,T86A, H105Y, E117K	0	1 (0.70%)	1.000
-35 inserted sequence[e], G45D	1 (1.69%)	0	0.292
-35 A to C[f], A39T	0	1 (0.70%)	1.000

[a] isolates with zoliflodacin MICs ≤0.002-0.015mg/L

[b] isolates with zoliflodacin MICs 0.125-0.25mg/L

[c] Determined by the χ^2 or Fisher exact test

[d] A (adenine) deletion in the 13-bp inverted-repeat sequence of the *mtrR* promoter.

[e] One isolate had an inserted 120 nucleotide-long sequence located at -35 in the mtrR promoter: TCAGA CGGCG CCCAT TCTTT AGGCG TTACC TTTTT CGCCC CCGTC ATACC GGCGA TACTG ATGCC TTCCA CAACC ACCTT GTCCC CGTCC TTCAG ACCCG ACGTA ACAAT CCAAT TCGTA C

[f] One isolate had a nucleotide substitution of A to C located at -35 in the *mtrR* promoter.
